# Characteristics of *Clostridium difficile* isolates and the burden of hospital-acquired *Clostridium difficile* infection in a tertiary teaching hospital in Chongqing, Southwest China

**DOI:** 10.1186/s12879-020-05014-6

**Published:** 2020-04-15

**Authors:** Wei Dai, Tianxiang Yang, Li Yan, Siqiang Niu, Chuanming Zhang, Jide Sun, Zhu Wang, Yun Xia

**Affiliations:** 1 Department of Laboratory Medicine, The First Affiliated Hospital of Chongqing Medical University, No.1 Youyi Road, Yuzhong District, Chongqing, 400016 People’s Republic of China; 2 Department of Laboratory Medicine, Dianjiang People’s Hospital of Chongqing, No.116 North Street, Guixi Street, Dianjiang County, Chongqing, 408300 People’s Republic of China

**Keywords:** Hospital-acquired *Clostridium difficile* infection, Risk factor, Genotyping, Antimicrobial resistance

## Abstract

**Background:**

*Clostridium difficile* infection (CDI), especially hospital-acquired *Clostridium difficile* infection (HA-CDI), continues to be a public health problem and has aroused great concern worldwide for years. This study aimed to elucidate the clinical and epidemiological features of HA-CDI and the characteristics of *C.difficile* isolates in Chongqing, Southwest China.

**Methods:**

A case-control study was performed to identify the clinical incidence and risk factors of HA-CDI. *C. difficile* isolates were characterised by polymerase chain reaction (PCR) ribotyping, multilocus sequence typing (MLST), toxin gene detection and antimicrobial susceptibility testing.

**Results:**

Of the 175 suspicious patients, a total of 122 patients with antibiotic-associated diarrhea (AAD) were included in the study; among them, 38 had HA-CDI. The incidence of AAD and HA-CDI was 0.58 and 0.18 per 1000 patient admissions, respectively. Chronic renal disease and cephalosporin use were independent risk factors for HA-CDI. Fifty-five strains were assigned into 16 sequence types (STs) and 15 ribotypes (RTs). ST2/RT449 (8, 14.5%) was the predominant genotype. Of the 38 toxigenic isolates, A + B + CDT- isolates accounted for most (34, 89.5%) and 1 A + B + CDT+ isolate emerged. No isolate was resistant to vancomycin, metronidazole or tigecycline, with A-B-CDT- being more resistant than A + B + CDT-.

**Conclusions:**

Different genotypes of *C. difficile* strains were witnessed in Chongqing, which hinted at the necessary surveillance of HA-CDI. Adequate awareness of patients at high risk of HA-CDI acquisition is advocated and cautious adoption of cephalosporins should be highlighted.

## Background

As a successful nosocomial pathogen, toxin-producing *C. difficile* has caused approximately 10–30% healthcare-associated infections [1, 2]. Increased incidence and severity of *Clostridium difficile* infection (CDI) have been witnessed in Europe and North America in recent decades [3, 4]. However, in developing countries, due to the poor awareness of healthcare workers and limited capacity of laboratory diagnosis, the potential public threat of CDI has not been fully recognized. A recent random-effects study including 37,663 patients reported a similar incidence rate of CDI in Asia in comparison with North America and Europe. Significant regional variation has been revealed and when compared with the Middle East and South Asia, East Asia was exposed to the highest CDI prevalence of 19.5% [5], which necessitated good awareness and surveillance of CDI in this area.

However, unlike the rest of East Asia, limited data have focused on the burden of CDI in China. Although few regional studies alarmed that the hyper-virulent *C. difficile* strain ST-1 (BI/NAP1/027), an epidemic strain in Europe and North America, has emerged in Chinese hospital settings, recent reports revealed that ST35, ST37 and ST3 were the most prevalent genotypes in mainland China [6, 7]. Moreover, in consideration of the complex personnel mobility in medical institutions, the majority of CDI is hospital-acquired, and nosocomial transmission of *C. difficile* contributes greatly to the spread of different genotypes. Recently, whole genome sequencing (WGS) identified the dissemination and spread of *C. difficile* ribotype 027 (RT027) and sequence type 081 (ST081) in two Chinese hospitals [8, 9]. Therefore, a better understanding of regional epidemiology is helpful to guide priorities for the management of hospital-acquired *Clostridium difficile* infection (HA-CDI). Although many studies have explored the CDI situation in China, the lack of epidemiological data in blind areas impedes a full understanding of CDI in this country. To the best of our knowledge, this is the first study of HA-CDI in Chongqing, a provincial administrative unit in Southwest China [6]. Our study was initiated to investigate the impact of HA-CDI by identifying its prevalence, determine the risk factors for the acquisition of this dilemma in patients with antibiotic-associated diarrhea (AAD), reveal the mortality of HA-CDI in this teaching hospital and inquire into the molecular epidemiology and antimicrobial resistance of *C.difficile* isolates found in this study.

## Methods

### Study design

**Fig. 1:**
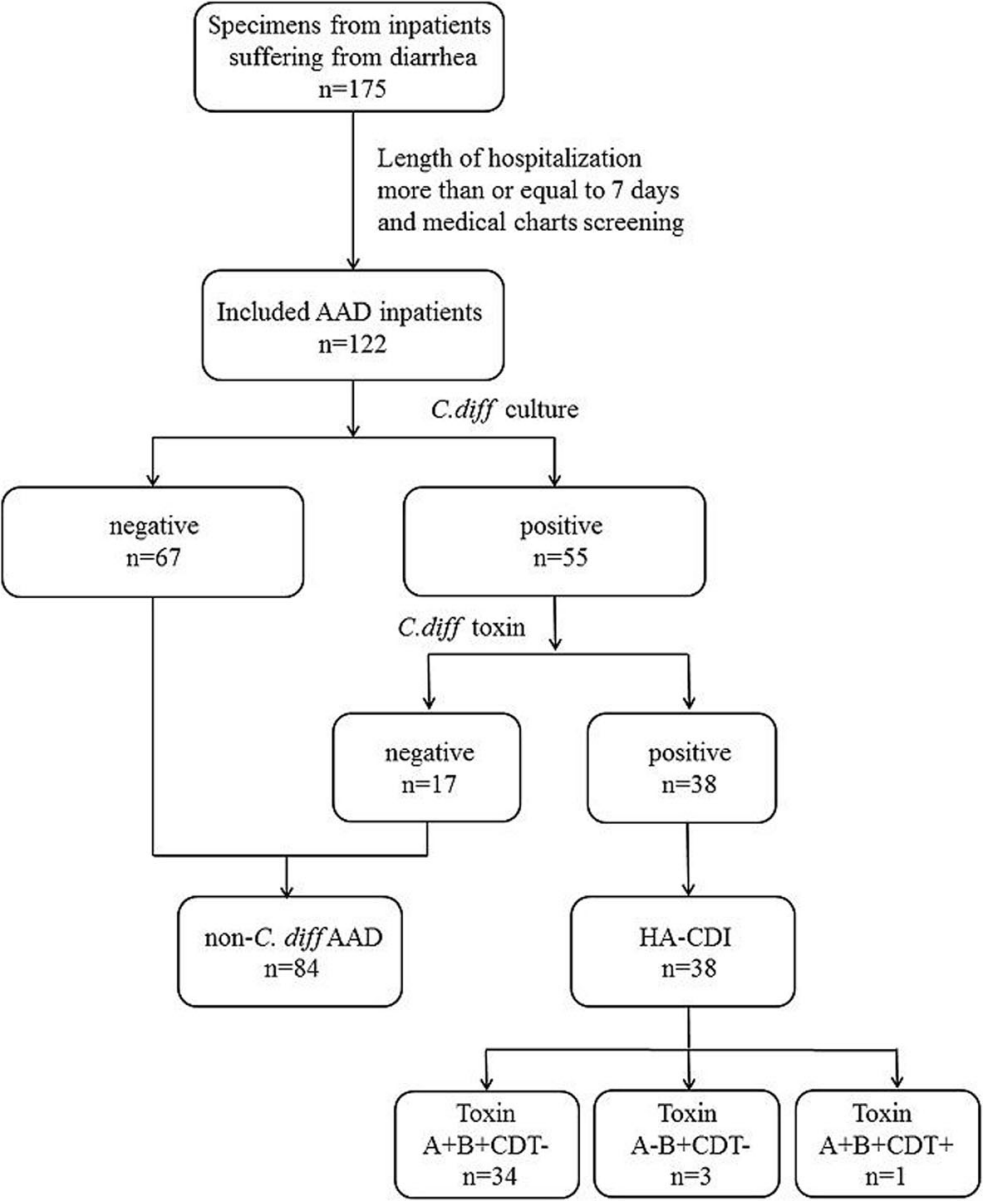
The flow diagram of data collection and laboratory diagnosis of *C. difficile* infection. Stool samples from patients with antibiotic-associated diarrhea (AAD) were cultured for *C. difficile*. Isolates from culture positive patients were assayed for toxin A/B in vitro and if positive for toxin in vitro, cases were identified as HA-CDI. Cases were further categorized by PCR amplification of isolates to determine their toxin gene profiles (toxin A, B, CDT). This surveillance was carried out from June 2014 to March 2016 in the First Affiliated Hospital of Chongqing Medical University

A case-control study was conducted from June 2014 to March 2016 in the First Affiliated Hospital of Chongqing Medical University, a tertiary teaching hospital with 3200 beds, which is the surveillance center of antimicrobial resistance in Chongqing. Unformed stools of inpatients suffering from diarrhea were collected for toxigenic culture of *C. difficile*. After medical chart screening, patients who were hospitalized for more than or equal to 7 days and who were administered antibiotics before diarrhea were included in this study. According to the results from toxigenic culture, patients diagnosed with HA-CDI were enrolled in the case group, while patients diagnosed with non*-C. difficile* AAD were enrolled in the control group (Fig. 1). Clinical data including demography, chronic underlying disease, comorbidities, medication prior to the onset of diarrhea, in-hospital recurrence and mortality were retrieved by electronic medical charts.

### Definitions

AAD was diagnosed when a hospitalized patient suffered from unexplained diarrhea in association with the administration of antibiotics during current hospitalization [1].

CDI was diagnosed when a patient with diarrhea was positive for a toxin-producing *C. difficile* strain in stool culture.

HA-CDI was the case with CDI confirmed at least 48 h after admission.

Four severity levels of CDI (mild, moderate, severe and complicated) introduced by Leffler et al. [10] were adopted to judge the clinical manifestations of patients in this study. Clinical outcomes of HA-CDI cases were classified into three categories: a. symptomatic recovery, b. CDI symptomatic recurrence while in hospital, c. in-hospital death after the diagnosis of HA-CDI.

### Microbiological testing

Unformed stool samples treated with alcohol shock were inoculated onto cycloserin-cefoxitin-fructose agar (CCFA) and cultured anaerobically in 37 °C for 5 days. The colonies suspected as *C. difficile* were subjected to mass spectrometry (Vitek MS, bioMerieux, France). *C. difficile* strains were assayed for toxin A and toxin B antigen by enzyme linked fluorescent assay (ELFA) (Vidas mini, bioMerieux, France) performed on culture supernatants in vitro. DNA of the *C. difficile* strain was extracted and polymerase chain reaction (PCR) was performed to detect the presence of toxin genes (*tcdA*, *tcdB*), the regulatory gene (*tcdC*) and binary toxin genes (*cdtA* and *cdtB*) as previously reported [11, 12]**.** The flow diagram of the laboratory diagnosis of *C. difficile* infection by toxigenic culture was summarized in Fig. 1. Antimicrobial susceptibilities of *C. difficile* to seven antibiotics (vancomycin, metronidazole, rifampin, levofloxacin, erythromycin, clindamycin and tigecycline) were tested by the agar dilution method as recommended by the Clinical and Laboratory Standards Institute (CLSI) M11-A8 [13]. Breakpoints for clindamycin and metronidazole were according to CLSI recommendations for anaerobic bacteria [14], while those for rifampin, erythromycin and levofloxacin were adopted on the basis of the suggestion made by Lidan C et al. [15]. Interpretation criteria of vancomycin and tigecycline were according to European Committee on Antimicrobial Susceptibility Testing (EUCAST) recommendations [16].

### Molecular typing analysis

Multilocus sequence typing (MLST) was performed by sequencing seven house-keeping genes of *C. difficile* (*adk, atpA, dxr, glyA, recA, sodA* and *tpi*) as previously reported by Griffiths D et al. [11]. Sequence types (STs) and clades of *C. difficile* strains were confirmed by querying on http://pubmlst.org/ website. A minimum spanning tree generated from BioNumerics version 7.6 was used to show the genetic diversity of the MLST data derived from this study.

Capillary gel electrophoresis-based PCR ribotyping was implemented according to a previous report by Fawley WN et al. [17]. Ribotypes were identified by querying on the WEBRIBO web-based database (http://webribo.ages.at). The novel ribotype was named as “Chongqing Ribotype” (CQR) plus two Arabic numbers (e.g., CQR01).

### Statistical analysis

Patients with a length of hospitalization more than or equal to 7 days were included in the statistical analysis. A univariate analysis was initially conducted to determine the potential risk factors for the acquisition of HA-CDI by comparing the HA-CDI group with the non- *C. difficile* AAD group. Categorical variables were compared by use of *chi-square* or *Fisher’s* exact test. Odds ratios (ORs) and 95% confidence intervals (CIs) were calculated to evaluate the strength of any association. Variables with a *P* value of < 0.10 in the univariate test were included in a multivariate one-step logistic remodel. A two-tailed *P*-value of < 0.05 was considered to be statistically significant. All of the statistical calculations were performed with standard programs in SPSS v.21.0 (SPSS, Chicago, IL, USA) [18].

## Results

### The prevalence of AAD and HA-CDI

During the surveillance period, a total of 211,536 patients were hospitalised and 91,800 received antibiotic treatment. A total of 175 patients developed diarrhea but only 122 (69.7%) received antibiotic treatment before that thus were included in this study as AAD patients. AAD developed in 1.3‰ of antibiotic-treated inpatients and had an incidence of 0.58 per 1000 patient admissions in this hospital. Among the 122 AAD patients, *C. difficile* was isolated from the specimens of 55 respondents, and 38 (31.1%) were positive for toxigenic *C. difficile* culture and diagnosed with HA-CDI, yielding an incidence of 0.41 HA-CDI per 1000 antibiotic-treated patients and 0.18 HA-CDI per 1000 patient admissions. Among the HA-CDI patients, the average age was 54.5 ± 17.4 years and 60.5% of the patients were older than 65 years. Twenty-eight (73.7%) were males. A majority of HA-CDI patients were from surgical wards. The median time between admission and the onset of diarrhea was 7 days, while that between admission and diagnosis of HA-CDI was 14 days. The severity of patients with HA-CDI ranged from mild to moderate, no severe HA-CDI case was recorded in this work. Thirty-five out of 38 (92.1%) HA-CDI patients had symptomatic recovery and no recurrence was noted. Three (7.9%) patients died during hospitalisation but not because of HA-CDI.

### Clinical characteristics and risk factors for HA-CDI

The clinical characteristics and risk factors for HA-CDI in AAD patients were summarized in Table 1. No significant demographic differences (such as age and gender) were observed between the two groups. Compared with the non-*C. difficile* AAD cases, HA-CDI patients were significantly more likely to have surgery in the last 6 months and more prone to suffering from chronic renal disease, pulmonary infection and hypoalbuminemia on admission. Exposure to chemotherapy, cephalosporins, metronidazole and proton pump inhibitors (PPIs) use were more frequent in patients with HA-CDI. Multivariate analysis showed that chronic kidney diseases (OR, 4.275; 95% CI, 1.154–15.839; *P* = .030) and cephalosporins exposure (OR, 8.840; 95% CI, 2.807–27.836; *P* = .000) were independent risk factors for HA-CDI acquisition in AAD patients.

**Table 1:** Statistical analysis for risk factors of hospital-acquired *C. difficile* infection (HA-CDI) in AAD patients

Variable	HA-CDI (*n* = 38) No.(%)	Non-*C. difficile* AAD (*n* = 84) No.(%)	Univariate analysis		Multivariate analysis	
			*P* value	OR	*P* value	OR(95%CI)
**Demographic data**						
Male gender	28(73.7)	62(73.8)	0.988	1.006		
Elderly(≥65 years)	23(60.5)	37(44.0)	0.795	0.949		
Admission to ICU	16(42.1)	38(45.2)	0.747	0.931		
**Comorbidities**						
Chronic kidney diseases	12(31.6)	12(14.3)	0.026*	2.211	0.030*	4.275 (1.154–15.839)
Coronary heart disease	3(7.9)	5(6.0)	0.703	1.326		
Diabetes mellitus	8(21.1)	16(19.0)	0.796	1.105		
Hypertension	13(34.2)	30(35.7)	0.872	0.958		
Hepatic disease	12(31.6)	15(17.9)	0.091	1.768		
Malignancy	7(18.4)	15(17.9)	0.940	1.032		
Surgery in the past 6 months	16(42.1)	16(19.0)	0.007*	2.211		
**Diagnosis on admission**						
Hypoalbuminaemia	25(65.8.)	16(19.0)	0.000*	3.454		
Urinary tract infection	5(13.2)	8(9.5)	0.541	1.382		
Pulmonary infection	22(57.9)	25(29.8)	0.003*	1.945		
Bloodstream infection	2(5.3)	7(8.3)	0.719	0.632		
**Medication prior to the onset of diarrhea during hospitalization**						
Glucocorticoids	12(31.6)	21(25.0)	0.449	1.263		
Chemotherapy	7(18.4)	3(3.6)	0.010*	5.158		
PPIs	20(52.6)	64(76.2)	0.009*	0.691		
Penicillin	17(44.7)	41(48.8)	0.677	0.917		
Cephalosporins	23(60.5)	10(11.9)	0.000*	5.084	0.000*	8.840 (2.807–27.836)
Carbapenems	16(42.1)	39(46.4)	0.677	0.907		
Aminoglycosides	5(13.2)	7(8.3)	0.513	1.579		
Fluoroquinolones	5(13.2)	15(17.9)	0.605	0.737		
Glycopeptides	15(39.5)	30(35.7)	0.690	1.105		
Metronidazole	13(34.2)	9(10.7)	0.020*	3.193		

*HA-CDI* hospital-acquired *C. difficile* infection, *AAD* antibiotic-associated diarrhea, *OR* odds ratio, *CI* confidence interval, *PPIs* Proton pump inhibitors, * *p* < 0.05

### Genotyping characteristics of *C. difficile* isolates

**Fig. 2:**
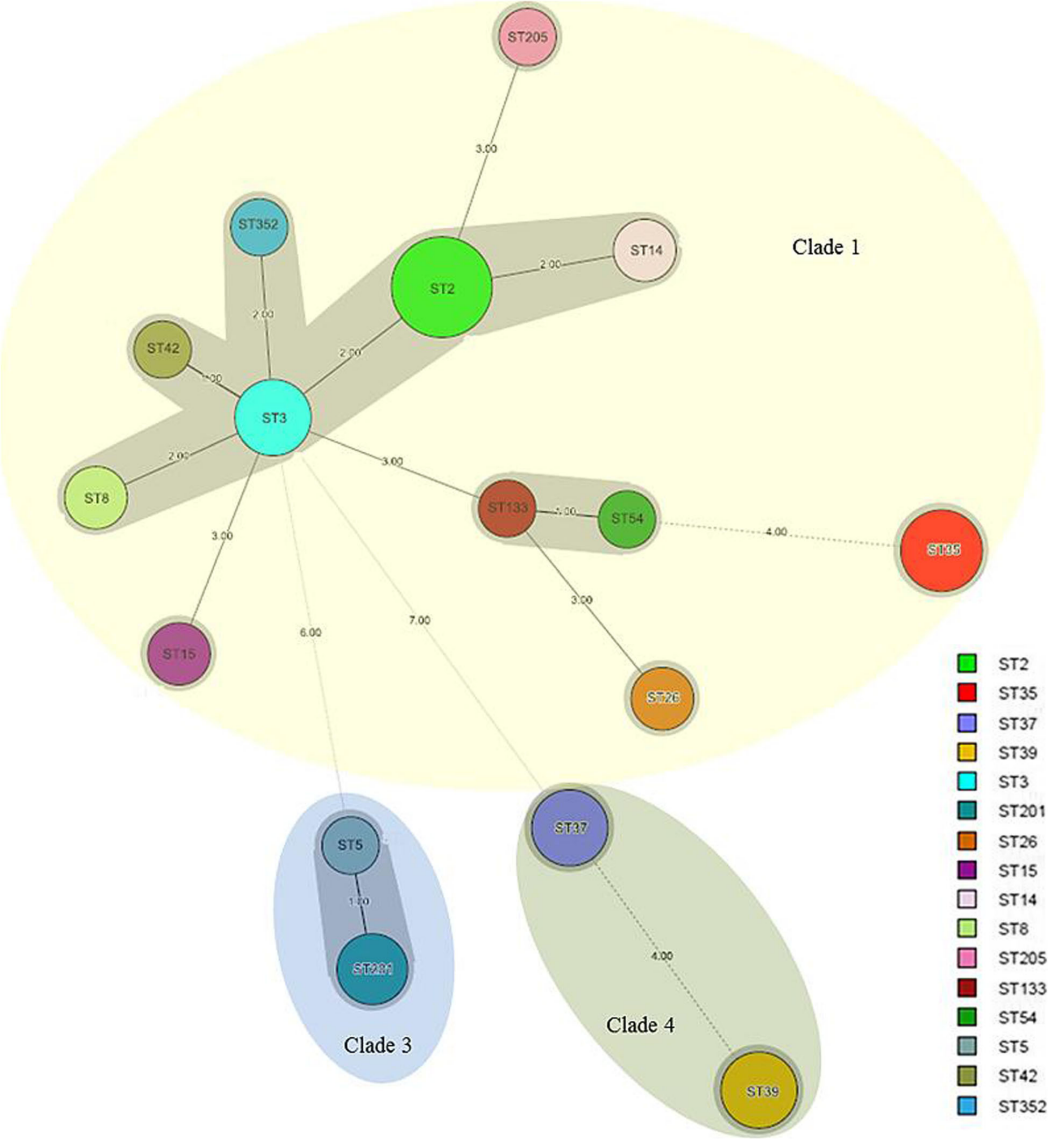
The minimum spanning tree for displaying the distribution and relationship of MLST sequence types found in this study. The circle size represented the number of isolates of each corresponding type. The figure on the line linking two circles demonstrated the number of different loci between them. The types with less than or equal to two different loci were covered by the gray area. Three clades were indicated by colourful areas

In total, 55 non-duplicated strains were isolated and assigned to 16 genotypes by MLST. ST2 (*n* = 9, 16.4%) was the most common genotype, followed by ST39 (*n* = 7, 12.7%) and ST37 (*n* = 6, 10.9%). ST35, ST54 and ST205 were commonly detected. A novel genotype ST352 was found. A majority (*n* = 40, 72.7%) of isolates were categorized as clade 1, followed by clade 4 (*n* = 13, 23.6%) and clade 3 (*n* = 2, 3.6%). The minimum spanning tree showed the relationship of ST types in Fig. 2.

**Fig. 3:**
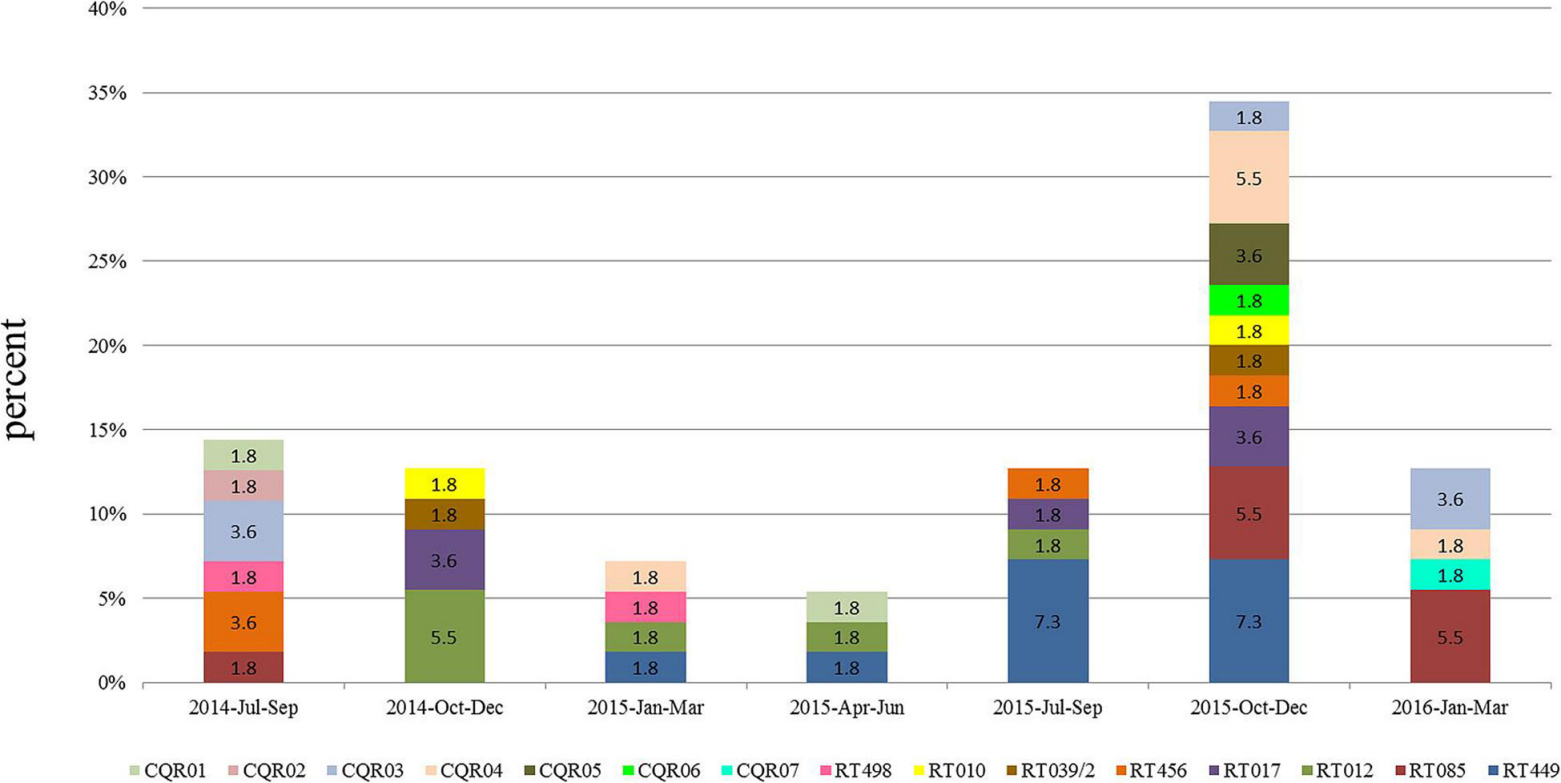
Seasonal superimposition of ribotypes among 55 *C. difficile* isolates from June 2014 to March 2016

All 55 isolates recovered were finally assigned to 15 PCR ribotypes (RTs) with 8 known RTs and 7 novel RTs. RT449 (*n* = 10, 18.2%), RT085 (*n* = 7, 12.7%), RT012 (*n* = 6, 10.9%), and RT017 (*n* = 5, 9.1%) were the main RTs. Of the seven novel ribotypes, CQR03 and CQR04 exhibited a high prevalence (*n* = 5, 9.1%). None of the isolates belonged to RT027 or RT078. Superimposition of the percentage diagram with the time of *C. difficile* detection by season revealed predominant proportions of RT449, RT085, and RT012 in this study (Fig. 3). Looking through the data derived from the two methods, ST2/RT449 (8, 14.5%) was the predominant genotype, followed by ST39/RT085 (7, 12.7%), ST54/RT012 (5, 9%) and ST37/RT017 (5, 9%). Exclusive correlations were found among three groups: ST26/RT39/2, ST39/RT085 and ST3/RT456.

### Toxigenic characteristics and their correlation with the genotypes of isolates

In all 55 isolates, an exclusive correlation was found between toxin types and genotypes (seen in the Additional file 1). All the ST39/RT085 isolates were A-B-, while all the RT012 isolates were toxin-producing (A + B+). Of the 55 isolates, 38 (69.1%) were toxin-producing, including 34 (89.5%) with toxigenic type A + B + CDT-, 3 (7.9%) with toxigenic type A-B + CDT- and 1 (2.6%) with toxigenic type A + B + CDT+.

Of the 38 toxigenic isolates, ST2/RT449 (8, 21.1%) and ST54/RT012 (5, 13.2%) were the predominant toxigenic genotypes. In MLST, ST2 (*n* = 9, 23.7%) was most frequently detected, followed by ST37 (*n* = 6, 15.8%), ST54 (*n* = 5, 13.2%) and ST35 (*n* = 5, 13.2%). PCR ribotyping found 11 ribotypes, including 6 known ribotypes and 5 novel ribotypes. RT449 (*n* = 9, 23.7%), RT012 (*n* = 6, 15.8%) and RT017 (*n* = 5, 13.2%) were the most frequent ribotypes. The CDT+ strain was assigned to the genotype of ST5/RT498 in clade 3 (Table 2).

**Table 2:** Typing results and toxin genotypes of 55 *C. difficile *isolates

Clades	MLST	CGE	*tcdA*	*tcdB*	*tcdC*	*cdtA*	*cdtB*	No.of isolates
1	ST8	CQR02	+	+	+	–	–	1
		RT010	+	+	+	–	–	1
	ST3	RT456	+	+	+	–	–	4
	ST42	CQR01	+	+	+	–	–	2
	ST54	RT012	+	+	+	–	–	5
	ST2	RT449	+	+	+	–	–	8
		CQR07	+	+	+	–	–	1
	ST35	CQR04	+	+	+	–	–	5
	ST133	RT449	+	+	+	–	–	1
	ST14	CQR05	+	+	+	–	–	2
	ST205	CQR03	–	–	–	–	–	5
	ST26	RT39/2	–	–	–	–	–	2
	ST15	RT449	–	–	–	–	–	1
		RT010	–	–	–	–	–	1
	ST352	CQR06	–	–	–	–	–	1
3	ST5	RT498	+	+	+	+	+	1
	ST201	RT498	+	+	+	–	–	1
4	ST37	RT017	–	+	+	–	–	3
		RT017	+	+	+	–	–	2
		RT012	+	+	+	–	–	1
	ST39	RT085	–	–	–	–	–	7

*MLST* multilocus sequence typing, *CGE* capillary gel electrophoresis, *CQR* Chongqing Ribotype New-ribo-type found in Chongqing, *ST* sequence type, *RT* ribotype

### Antimicrobial susceptibility between genotypes and toxin types of isolates

The minimum inhibitory concentrations (MICs) of seven antimicrobial agents for 55 non-duplicated strains were summarized in Table 3 (raw data shown in Additional file 2). Eight (14.5%) isolates were found to be multidrug resistant (MDR). None of the isolates were resistant to vancomycin, metronidazole or tigecycline, while high resistance to erythromycin and clindamycin was observed with rates of 87.3 and 61.8%, respectively. Twelve point seven percent of the isolates were resistant to rifampin and 14.5% were resistant to levofloxacin.

**Table 3:** The drug susceptibility results and MIC ranges of all the 55 *C. difficile* isolates

Antimicrobial agent	Resistance breakpoints and ECOFFs (ug/ml)	All strains(*n* = 55)				A + B+ strains(*n* = 35)				A-B- strains(*n* = 17)				A-B + strains(n = 3)			
		MIC (ug/ml)			% of isolates	MIC (ug/ml)			% of isolates	MIC (ug/ml)			% of isolates	MIC (ug/ml)			% of isolates
		MIC_50_	MIC_90_	Range	R	MIC_50_	MIC_90_	Range	R	MIC_50_	MIC_90_	Range	R	MIC_50_	MIC_90_	Range	R
Vancomycin^a^	> 2	0.5	0.5	0.125–1	0	0.5	0.5	0.5–1	0	0.5	0.5	0.25–0.5	0	0.5	0.5	0.125–0.5	0
Metronidazole^b^	> = 32	0.25	0.25	0.125–1	0	0.25	0.25	0.125–1	0	0.125	0.25	0.125–0.25	0	0.125	0.25	0.125–0.25	0
Rifampin^c^	> = 8	0.0625	256	0.0625–512	12.7	0.0625	0.0625	0.0625–512	2.9	256	512	0.0625–512	66.7	0.0625	512	0.0625–512	23.5
Levofloxacin^c^	> = 8	2	128	0.5–128	14.5	2	2	0.5–128	2.9	8	128	2–128	66.7	2	128	0.5–128	29.4
Erythromycin^c^	> = 8	256	512	2–512	87.3	256	512	2–512	82.9	512	512	512–512	100	64	512	2–512	94.1
Clindamycin^b^	> = 8	16	256	0.5–256	61.8	8	256	0.5–256	57.1	256	256	128–256	100	128	256	0.5–256	64.7
Tigecycline^d^	> 0.25	0.0625	0.0625	0.0625–0.0625	0	0.0625	0.0625	0.0625–0.0625	0	0.0625	0.0625	0.0625–0.0625	0	0.0625	0.0625	0.0625–0.0625	0

*MIC* minimum inhibitory concentration, ^a^For vancomycin, MIC breakpoint is recommended by the EUCAST (2019); ^b^The applied MIC breakpoints are those recommended for anaerobes by the CLSI (2019), M100 29th ed.; ^c^Breakpoints were suggested by Chen Lidan et al. [14]; ^d^For tigecycline, MICs were compared to the EUCAST (2019) epidemiological cut-off value (0.25 μg/ml)

In comparison with A + B+ isolates and A-B+ isolates, higher resistance rates of A-B- isolates to rifampin, levofloxacin, erythromycin and clindamycin were revealed. Relatively low resistance rates of A + B+ isolates to rifampin (2.9%) and levofloxacin (2.9%) were observed.

Varied antimicrobial phenotypes demonstrated in different RTs. The resistance rates of RT012 to erythromycin and clindamycin (100 and 83.3%, respectively) were higher than those of RT449. All of the RT017 isolates were co-resistant to erythromycin and clindamycin. Sixty percent of the RT017 isolates were co-resistant to erythromycin, clindamycin and levofloxacin.

## Discussion

Enhanced molecular diagnostic and antibiotic treatment strategies promote the continuous evolution of the knowledge of CDI epidemiology. Geographical heterogeneity and transcontinental dissemination have aroused more concerns about regional CDI surveillance. In China, although the state of dilemma introduced by CDI has been documented before [6, 7], data are lacking in the central and western regions. To fill gaps in the epidemiological territory of CDI in China, the results in this study presented basic knowledge of the prevalence and mortality of HA-CDI, and helped to improve the recognition of patients at high risk for HA-CDI acquisition and to guide antibiotic stewardship initiatives of HA-CDI in this tertiary teaching hospital in Southwest China.

A survey focusing on antibiotic consumption in specialized public hospitals in 30 provinces in mainland China showed a decrease in the percentage of antibiotic use in inpatients in Chongqing, from 78.84 to 54.93% [19]. The present study found the ratio of antibiotic use in inpatients was 43.4%. Despite being relatively low and comparable to the previous data reported by Zhou et al. [20], this percentage, to a large extent, surpassed the recommendation of 30% by World Health Organization (WHO). Previous studies have reported varied frequencies of AAD from 0.57 to 14.9% in different populations [21, 22]. The correlation between antibiotic use and the prevalence of AAD in Chongqing was previously unknown. The present study witnessed a moderate prevalence of AAD in 0.13% (a much lower rate) of antibiotic-treated inpatients. One possible explanation is that a majority of the patients in this cohort were from surgical wards and received antibiotics simply for perioperative prophylaxis.

In this investigation, HA-CDI accounted for 31.1% of AAD, which was consistent with previous reports [20, 23]. The high prevalence of CDI among AAD is always a major concern worldwide [24]. To prevent CDI from AAD, external interventions and internal defense mechanisms should work cooperatively. Our previous study has shown that interleukin-27 (IL-27)/IL-27 receptor signaling provides protection against *C. difficile*-induced colitis in AAD patients [25]. A recent systematic review and meta-analysis reported an incidence of 0.32 cases of CDI per 1000 patient admissions in Asia [5] and a similar result was verified in a 7-year retrospective study in a large university hospital in Eastern China [26]. This study reported a relatively low incidence of 0.18 per 1000 patient admissions, probably due to inadequate awareness of CDI among clinicians, low sensitivity of stool anaerobic culture for *C. difficile* detection, and low testing frequency [27]. Another possible reason is the missing information of a proportion of inpatients who might develop CDI after discharge.

As is well known, the use of antibiotics may cause CDI [28], but the case-control design focusing on the difference between antibiotic group and non-antibiotic group may have the trends to overestimate the impact of antibiotic exposure on the acquisition of HA-CDI. To explore the specific reasons leading to CDI, this study set up a comparison between the HA-CDI group and the non-*C. difficile* AAD group in AAD patients to identify which antibiotics or predictors were associated with a high risk for HA-CDI. Although many risk factors were revealed in univariate analysis, only two independent risk factors, cephalosporin use prior to the onset of diarrhea and chronic kidney diseases were identified for patients with HA-CDI when compared to non-*C.difficile* AAD, which is consistent with previous reports [29–31] and not difficult to explain. In addition to the cephalosporins known to all [32], chronic renal disease may cause poor excretion of antibiotic agents, high concentration in blood and finally, the imbalance of bacterial flora in the gut.

Age over 65 years was not associated with an increased risk for HA-CDI. Suffering from HA-CDI in younger age was observed in this study. Similar results have been reported in several previous studies in mainland China and France [23, 29, 33]. Therefore, it is necessary to consider the age threshold in the recognition of inpatients at high risk for HA-CDI in different settings. Massive consumption of antibiotics by university students has been reported nationwide in China [34]. Accumulative effects of antibiotic consumption may contribute partly to the acquisition of CDI in younger age, which deserves more attention. Moreover, ageing is accompanied by changes in the gut microbiome [35], and it is speculated that it is not the age threshold, but the gut microbial structure that truly participates in the priming of HA-CDI.

To reveal the epidemiology of CDI in mainland China, in this non-outbreak situation, specific genotypes of toxigenic *C. difficile* strains were observed. ST2 was the most predominant genotype, while recent studies reported that ST54, ST3 and ST37 were the most prevalent genotypes in mainland China [6, 33, 36–38]. Noteworthily, in addition to ST54 and ST3, ST35 also emerged both in this work and another inspection in Yunnan [39], a province bordering Chongqing, witnessing the spread of this toxin genotype over provinces in China. Toxigenic RT449 with a high prevalence in this work was not reported previously, and its predominant proportion may indicate an upcoming outbreak. One *C. difficile* isolate was positive for binary toxin and belonged to ST5/RT498 in clade 3. Although the CDT+ strain appeared less frequently in Asia, this was not the first report of this toxin genotype in China. ST5 accounted for 83.7% of binary toxin gene-positive strains in a survey conducted by Chen et al. [40] in 2018. Data on *C. difficile* strains in clade 3 with binary toxins are not well documented in mainland China. WGS of three clade 3 *C. difficile* strains carrying binary toxin genes in a university hospital found that clade 3 has unusual clade-specific *PaLoc* characteristic of Tn*6218* insertion, which may be the main feature to distinguish clade 3 from other *C. difficile* [41]. The identification of seven novel RTs indicated the diversity of *C. difficile* strains in this hospital.

Despite the fact that clinical *C. difficile* strains with hetero-resistance or high-level resistance to metronidazole were reported in China [20, 33], the present study failed to identify strains resistant to metronidazole, vancomycin or tigecycline, indicating that these three antibiotic agents still seem to be appropriate for empirical treatment of HA-CDI. In addition, toxin types were associated with antibiotic resistance phenotypes. A-B- strains were more resistant than A + B+ strains, while the latest data from two hospitals in Shangdong illustrated that non-toxigenic strains were more sensitive [42].

Our study has some limitations. First, these results were derived from a single-center. Widely recommended detection schemes, two-step and three-step methods for the diagnosis of CDI, were implemented in many laboratories in China, but anaerobic culture was not the choice for the final confirmation. This may be one of the causes for the lack of epidemiological data in this country. To obtain surveillance data for CDI, a network of reference or central laboratories such as that found in Europe is needed [43]. Second, this study failed to track clinical treatments of HA-CDI, but most patients recovered from diarrhea after the discontinuation of antibiotic therapy. Third, highly sensitive tests, such as the nucleic acid amplification test (NAAT) or the glutamate dehydrogenase (GDH) screening test, were not performed in this study.

## Conclusions

In summary, this study presented a comprehensive survey of HA-CDI and AAD in Chongqing, Southwest China. The burdens of HA-CDI and AAD were moderate. Inpatients undergoing cephalosporins therapy and suffering from chronic kidney diseases, who are thus at high risk for HA-CDI, deserve more attention. The regional diversity of *C. difficile* strains in genotype necessitates good awareness of HA-CDI by holding an evolving insight into the surveillance of this adverse event. In addition to the notorious genotypes, sufficient attention should be paid to the relatively rare toxigenic strains found in this report, such as ST5/RT498, during molecular epidemiology monitoring.

## Supplementary information

**Additional file 1 **The detection results and department sources of 55 *C. difficile* strains.

**Additional file 2.** The MICs of seven antimicrobial agents for isolates included in this study.

## Data Availability

The datasets generated and analysed during the current study are included in this published article and its supplementary information files. More datasets are not publicly available due to the need for further reaserch, but are available from the corresponding author on reasonable request.

## Abbreviations

CDI: *Clostridium difficile* infection
HA-CDI: Hospital-acquired *Clostridium difficile* infection
AAD: Antibiotic-associated diarrhea
PCR: Polymerase Chain Reaction
RT: Ribotype
MLST: Multilocus sequence typing
CCFA: Cycloserin-cefoxitin-fructose agar
ELFA: Enzyme linked fluorescent assay
CLSI: Clinical and Laboratory Standards Institute
EUCAST: European Committee on Antimicrobial Susceptibility Testing
ST: Sequence type
CQR: Chongqing Ribotype
OR: Odds ratio
CI: Confidence interval
PPIs: Proton pump inhibitors
MIC: Minimum inhibitory concentration
MDR: Multidrug resistant
WHO: World health organization
IL: Interleukin
CGE: Capillary gel electrophoresis
WGS: Whole genome sequencing
NAAT: Nucleic acid amplification test
GDH: Glutamate dehydrogenase
