# Supplementary material for: Characteristics of Clostridium difficile isolates and the burden of hospital-acquired Clostridium difficile infection in a tertiary teaching hospital in Chongqing, Southwest China
Source: BMC Infect Dis. 2020 Apr 15;20:277. doi: 10.1186/s12879-020-05014-6 (PMC7157987; doi:10.1186/s12879-020-05014-6)
Supplement: Supplementary file 1 — Additional file 1 The detection results and department sources of 55 C. difficile strains. [file 12879_2020_5014_MOESM1_ESM.docx]

**Additional file 1** The detection results and department sources of 55 *C. difficile* strains

| Number | Genotyping | | *tcdA* | *tcdB* | *tcdC* | Binary toxin | | toxin^a^ | toxin^b^ | Clinical department |
| --- | --- | --- | --- | --- | --- | --- | --- | --- | --- | --- |
|  | CGE | MLST |  |  |  | *cdtA* | *cdtB* |  |  |  |
| 1 | RT498 | ST5 | + | + | + | + | + | 0.41 | 0.46 | ICU |
| 2 | CQR02 | ST8 | + | + | + | - | - | 1.44 | 0.89 | Neurosurgery |
| 3 | RT456 | ST3 | + | + | + | - | - | 0.46 | 0.52 | Gastrointestinal surgery |
| 4 | CQR01 | ST42 | + | + | + | - | - | 3.01 | 1.78 | ICU |
| 5 | RT085 | ST39 | - | - | - | - | - | 0.01 | 0.02 | Gastrointestinal surgery |
| 6 | CQR03 | ST205 | - | - | - | - | - | 0.03 | 0.04 | ICU |
| 7 | RT012 | ST54 | + | + | + | - | - | 1.56 | 0.89 | Neurosurgery |
| 8 | RT012 | ST54 | + | + | + | - | - | 0.83 | 0.93 | Neurosurgery |
| 9 | RT012 | ST54 | + | + | + | - | - | 0.63 | 0.78 | ICU |
| 10 | CQR03 | ST205 | - | - | - | - | - | 0.11 | 0.08 | Respiratory medicine |
| 11 | RT456 | ST3 | + | + | + | - | - | 5.32 | 1.74 | Gastrointestinal surgery |
| 12 | RT039/2 | ST26 | - | - | - | - | - | 0.02 | 0.03 | Gastroenterology |
| 13 | RT017 | ST37 | - | + | + | - | - | 5.11 | 0.63 | Respiratory medicine |
| 14 | RT017 | ST37 | - | + | + | - | - | 1.39 | 0.58 | ICU |
| 15 | RT010 | ST8 | + | + | + | - | - | 1.62 | 1.45 | Neurosurgery |
| 16 | RT449 | ST2 | + | + | + | - | - | 0.64 | 0.89 | Neurosurgery |
| 17 | CQR04 | ST35 | + | + | + | - | - | 0.50 | 0.59 | Neurology |
| 18 | CQR03 | ST205 | - | - | - | - | - | 0.03 | 0.02 | Neurosurgery |
| 19 | RT012 | ST54 | + | + | + | - | - | 0.52 | 0.46 | Neurosurgery |
| 20 | CQR01 | ST42 | + | + | + | - | - | 3.45 | 4.44 | Neurosurgery |
| 21 | RT017 | ST37 | - | + | + | - | - | 2.57 | 4.34 | ICU |
| 22 | RT449 | ST133 | + | + | + | - | - | 1.66 | 0.13 | ICU |
| 23 | RT449 | ST15 | - | - | - | - | - | 0.17 | 0.03 | Neurology |
| 24 | RT449 | ST2 | + | + | + | - | - | 2.46 | 0.78 | Neurosurgery |
| 25 | RT012 | ST37 | + | + | + | - | - | 3.84 | 0.24 | Urinary surgery |
| 26 | RT449 | ST2 | + | + | + | - | - | 4.53 | 4.02 | Nephrology |
| 27 | RT449 | ST2 | + | + | + | - | - | 0.78 | 0.65 | Urinary surgery |
| 28 | RT456 | ST3 | + | + | + | - | - | 0.54 | 0.43 | Geriatrics |
| 29 | RT449 | ST2 | + | + | + | - | - | 3.78 | 1.46 | ICU |
| 30 | RT010 | ST15 | - | - | - | - | - | 0.04 | 0.01 | Geriatrics |
| 31 | RT012 | ST54 | + | + | + | - | - | 0.87 | 1.04 | Emergency medicine |
| 32 | RT498 | ST201 | + | + | + | - | - | 0.39 | 0.44 | Neurosurgery |
| 33 | RT085 | ST39 | - | - | - | - | - | 0.04 | 0.07 | Neurology |
| 34 | RT085 | ST39 | - | - | - | - | - | 0.01 | 0.01 | Neurosurgery |
| 35 | RT449 | ST2 | + | + | + | - | - | 0.43 | 0.59 | Gastroenterology |
| 36 | RT449 | ST2 | + | + | + | - | - | 0.45 | 0.32 | Gastroenterology |
| 37 | RT456 | ST3 | + | + | + | - | - | 1.20 | 1.81 | Oncology |
| 38 | RT449 | ST2 | + | + | + | - | - | 0.44 | 0.40 | Infectious disease |
| 39 | CQR05 | ST14 | + | + | + | - | - | 1.57 | 3.33 | Nephrology |
| 40 | CQR04 | ST35 | + | + | + | - | - | 0.74 | 0.63 | Hepatobiliary surgery |
| 41 | CQR04 | ST35 | + | + | + | - | - | 0.65 | 0.95 | Cardiology |
| 42 | RT039/2 | ST26 | - | - | - | - | - | 0.04 | 0.02 | Hematology |
| 43 | CQR06 | ST352 | - | - | - | - | - | 0.02 | 0.01 | Respiratory medicine |
| 44 | RT017 | ST37 | + | + | + | - | - | 0.67 | 1.67 | Gastroenterology |
| 45 | RT017 | ST37 | + | + | + | - | - | 1.53 | 2.56 | ICU |
| 46 | RT085 | ST39 | - | - | - | - | - | 0.01 | 0.02 | Nephrology |
| 47 | CQR04 | ST35 | + | + | + | - | - | 0.64 | 0.60 | Gastroenterology |
| 48 | CQR05 | ST14 | + | + | + | - | - | 1.04 | 3.33 | Nephrology |
| 49 | RT085 | ST39 | - | - | - | - | - | 0.03 | 0.01 | Cardiology |
| 50 | CQR07 | ST2 | + | + | + | - | - | 1.24 | 3.25 | Hematology |
| 51 | RT085 | ST39 | - | - | - | - | - | 0.01 | 0.03 | Infectious diseases |
| 52 | RT085 | ST39 | - | - | - | - | - | 0.01 | 0.01 | Rehabilitation |
| 53 | CQR03 | ST205 | - | - | - | - | - | 0.02 | 0.03 | Geriatrics |
| 54 | CQR03 | ST205 | - | - | - | - | - | 0.04 | 0.03 | Neurology |
| 55 | CQR04 | ST35 | + | + | + | - | - | 0.76 | 0.38 | Infectious diseases |

CGE capillary gel electrophoresis, MLST multilocus sequence typing, CQR Chongqing Ribotype,

New-ribo-type found in Chongqing, ICU intensive care unit, ^a^The direct toxin test on stool, the

cutoff value was 0.37; ^b^The toxin test on strain, the cutoff value was 0.13
